# Extensor tendon rupture caused by instability of the ulnar head with an osteoarthritic distal radioulnar joint: a case report

**DOI:** 10.1186/1752-1947-7-281

**Published:** 2013-12-30

**Authors:** Chul-Hyun Cho, Si-Wuk Lee

**Affiliations:** 1 Department of Orthopaedic Surgery, Dongsan Medical Center, School of Medicine, Keimyung University, 56, Dalseong-ro, Jung-gu, Daegu 700-712, South Korea

**Keywords:** Extensor tendon, Distal radioulnar joint, Rupture, Osteoarthritis, Instability, Scallop sign

## Abstract

**Introduction:**

Although spontaneous extensor tendon rupture often occurs in association with rheumatoid arthritis, extensor tendon rupture associated with osteoarthritis of the distal radioulnar joint has been rarely reported.

**Case presentation:**

We present the case of a 74-year-old Asian woman with a fourth and fifth extensor tendon rupture caused by instability of the ulnar head associated with an osteoarthritic distal radioulnar joint. Intraoperative findings showed that the cause of the dorsal capsular perforation and extensor tendon rupture was mechanical friction with the unstable ulnar head, which had no osteophytes or roughness. After tendon transfer and resection of the ulnar head, our patient can extend her ring and little fingers without difficulty for her daily activities.

**Conclusions:**

When a patient with osteoarthritic distal radioulnar joint has instability of the ulnar head and the ‘scallop sign’ on radiography, physicians should consider the possibility of extensor tendon rupture as a complication.

## Introduction

Although spontaneous extensor tendon rupture often occurs in association with rheumatoid arthritis [1,2], extensor tendon rupture associated with osteoarthritis of the distal radioulnar joint (DRUJ) has been rarely reported [3-8]. According to the previous literature, the mechanism of extensor tendon rupture results from the perforation of the dorsal capsule of the DRUJ by attrition of the posteriorly dislocated or subluxated ulnar head and osteophytes during pronation and supination [3,5]. Yamazaki and colleagues suggested that deepening and widening of the sigmoid notch, and radial shift of the ulnar head are radiologic risk factors for extensor tendon ruptures in patients with osteoarthritic DRUJ [8].

Here, we present a case of extensor tendon rupture caused by instability of the ulnar head with an osteoarthritic DRUJ. We discuss its pathomechanism and risk signs and conduct a literature review.

## Case presentation

A 74-year-old right-handed Asian woman was referred to our hospital with complaints of limited motion of her right little and ring fingers. Our patient had been experiencing an inability to extend her right little finger for two months, and subsequently, her ring finger for two weeks. Our patient denied any associated pain and warning symptoms such as tendon irritation.

**Figure 1:**
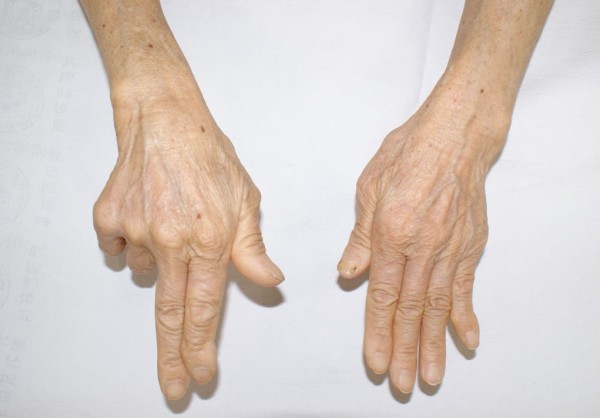
This 74-year-old right-handed woman was unable to actively extend her right little and ring fingers at the metacarpophalangeal joint.

**Figure 2:**
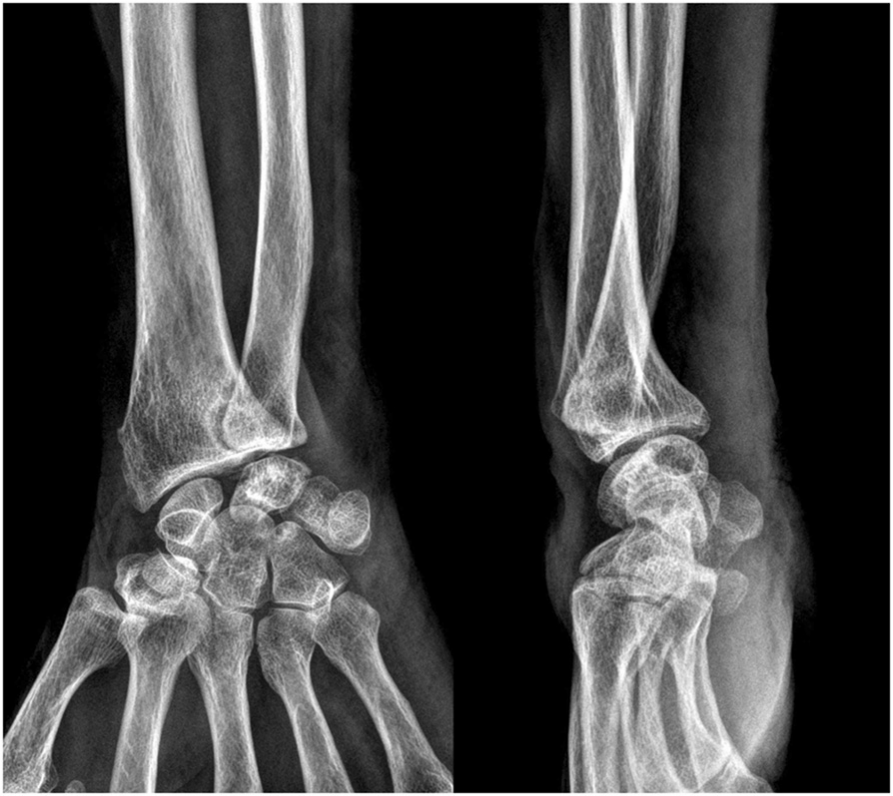
Plain radiographs show osteoarthritic change at the distal radioulnar joint with deepening and widening of the sigmoid notch.

On physical examination, our patient was unable to actively extend her right little and ring fingers at the metacarpophalangeal joint (Figure 1). A palpable mass with swelling at the wrist dorsum and severe instability of the DRUJ on the load and shift test was observed. Laboratory examinations revealed that her blood cell count, erythrocyte sedimentation rate, and C-reactive protein were within normal range. Her rheumatoid factor was also negative. Plain radiographs showed osteoarthritic change at the DRUJ. Deepening and widening of the sigmoid notch and radial shift of the ulnar head were also revealed (Figure 2).

**Figure 3:**
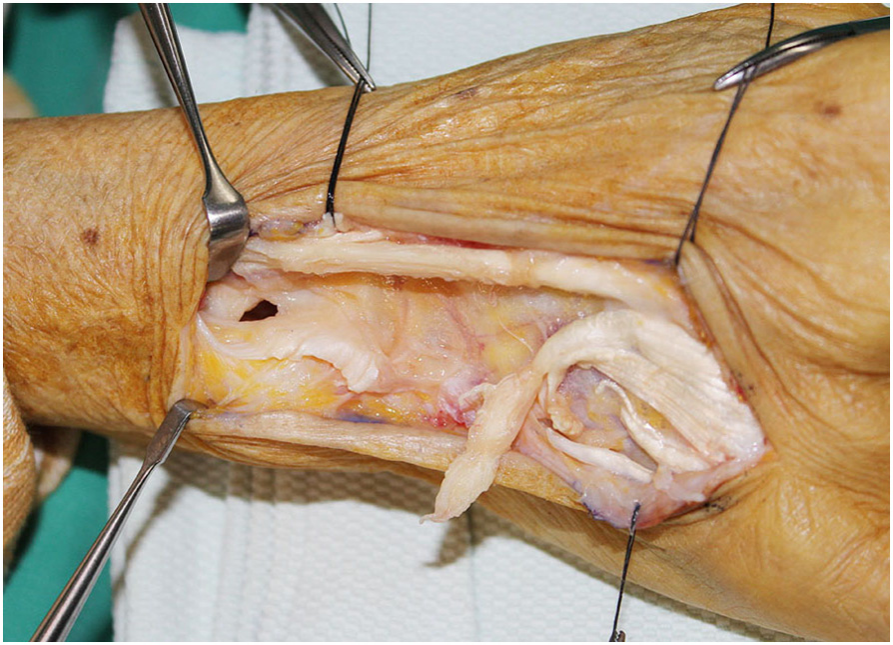
**An intraoperative photograph shows extensor digiti minimi, as well as the fourth and fifth extensor digitorum communis tendons, are ruptured with fraying of the tissue in the ruptured margin.** The ulnar head is directly exposed and the dorsally overlaying capsule is worn out.

Intraoperatively, extensor digiti minimi (EDM), along with the fourth and fifth extensor digitorum communis (EDC) tendons, were ruptured with fraying of the tissue in the ruptured margin. Their distal ends were matted together in the scar and the fraying tissue and their proximal ends lay just proximal to the DRUJ. The ulnar head was directly exposed and the dorsally overlaying capsule was worn out (Figure 3). The ulnar head had arthritic change, but a smoothened surface without osteophyte formation. Although mild synovitis was present, there was no macroscopic evidence of rheumatoid arthritis.

Because end-to-end repair was impossible, the extensor indicis proprius tendon was transferred to the EDM tendon, and the distal stumps of the fourth and fifth EDC tendon were sutured to the intact EDC tendon, in the end-to-side fashion. The ulnar head was resected. At 15 months after surgery, she can extend her ring and little fingers without difficulty for daily activities. She denied any pain around her wrist area.

## Discussion

Nontraumatic osteoarthritis of the DRUJ is uncommon and occurs principally in elderly patients [6]. Although extensor tendon rupture associated with osteoarthritis of the DRUJ has been rarely reported, it is a clinically important complication [3-8]. In 1948, Vaughan-Jackson first reported two cases of extensor tendon rupture by attrition at the osteoarthritic DRUJ [7]. Typically, extensor tendon rupture is sequential and begins from the little finger, followed by the ring and long fingers [4]. It may occur by attrition between the extensor tendon and posteriorly displaced ulnar head with osteophytes, which protrudes through a perforation of the dorsal capsule of the DRUJ [3,4].

Commonly reported radiographic findings in cases of osteoarthritic DRUJ combined with extensor tendon rupture included deepening and widening of the sigmoid notch, radial shift of the ulnar head, ulnar plus variance, osteophytes of the distal end of the ulna, and dorsal subluxation or dislocation of the ulnar head. Freiberg and Weinstein have termed the plain radiographic finding, a scalloping of the ulnar aspect of the distal radius, the ‘scallop sign’ [1]. The scallop sign is characterized by a sclerotic border with deepening and widening of the sigmoid notch. Beyond incidental interest, attention is called to the scallop sign because it seems diagnostic of existing or impending extensor tendon rupture [1]. Osteophytes of the radial aspect of the ulnar head and dorsal shift of the ulnar head may perforate the DRUJ capsule and allow abrasion of the extensors. Osteophytes in the DRUJ make contact with, and may abrade, the extensor tendon of the little finger, which has an ulnar location in the extensor tendon sheath. Ohshio and colleagues reported five cases of spontaneous rupture of the extensor tendon due to osteoarthritis of the DRUJ [4]. Several reports have described that the distal end of the ulna showed the plus variant, as well as dorsal dislocation or subluxation. Carr and Burge reported that perforation of the dorsal capsule of the DRUJ, allowing contact between the roughened ulnar head and extensor tendons, was present in all cases [3]. They described that the consistent finding of capsular perforation with protrusion of the roughed ulnar head is strong evidence in favor of attrition as a mechanism of tendon rupture in osteoarthritis. In our case, we observed a definite scallop sign, a radial shift of the ulnar head, and a smoothened ulnar head. Indeed, we could not find a dislocated or subluxated ulnar head, ulnar positive variance, osteophytes or roughness of the ulnar head on plain radiographs. Our operative findings showed that the cause of the dorsal capsular perforation and extensor tendon rupture was mechanical friction with the unstable ulnar head, which had no osteophytes or roughness.

Yamazaki and colleagues analyzed the radiographic morphology of the DRUJ to identify the risk factors for extensor tendon rupture [8]. They reported that the risk factors include severe osteoarthritic changes, exceeding Kellgren-Lawrence grade 3, deepening and widening of the sigmoid notch, radial shift of the ulnar head and dorsal inclination of the sigmoid notch. There was no significant association between tendon rupture and the morphology of the ulnar head or positive variance [8]. Our case is consistent with these findings. It means that extensor tendon rupture associated with osteoarthritic DRUJ can occur without osteophytes, dislocation or subluxation, and with a positive variance of the ulnar head on radiographic findings.

Treatment options for extensor tendon rupture with osteoarthritic DRUJ included end-to-end repair, tendon graft, and tendon transfer [4,5]. End-to-end repair is usually impossible because ruptured stumps have scars and fraying tissue. Tendon transfer is more preferable than tendon graft because it is technically easier and has a lower complication rate [4,5]. Excision of the distal ulna is necessary to prevent the recurrence of tendon rupture [4].

Although dorsal wrist pain or pain on active finger extension may precede tendon rupture in rheumatoid disease, it appears that there are few predictive or warning symptoms in osteoarthritic DRUJ [3]. In our case, there was no associated pain and warning symptoms, such as tendon irritation.

## Conclusions

When a patient with osteoarthritic DRUJ has instability of the ulnar head and a ‘scallop sign’ on radiography, physicians should consider the possibility of extensor tendon rupture as a complication.

## Consent

Written informed consent was obtained from the patient for publication of this case report and any accompanying images. A copy of the written consent is available for review by the Editor-in-Chief of this journal.

## Competing interests

All authors declare that they have no competing interests.

## Authors’ contributions

SWL reviewed and interpreted the patient data and X-ray. CHC performed the operation and was a major contributor in writing the manuscript. All authors read and approved the final manuscript.

## References

[B1] Freiberg RA Weinstein A The scallop sign and spontaneous rupture of finger extensor tendons in rheumatoid arthritis Clin Orthop Relat Res 1972 83 128 130 5014802 10.1097/00003086-197203000-00024

[B2] Gong HS Lee JO Baek GH Kim BS Kim JY Lee JS Song CH Extensor tendon rupture in rheumatoid arthritis: a survey of patients between 2005 and 2010 at five Korean hospitals Hand Surg 2012 17 43 47 10.1142/S0218810412500074 22351532

[B3] Carr AJ Burge PD Rupture of extensor tendons due to osteoarthritis of the distal radio-ulnar joint J Hand Surg 1992 17B 694 696 10.1016/0266-7681(92)90203-e 1484257

[B4] Ohshio I Ogino T Minami A Kato H Miyake A Extensor tendon rupture due to osteoarthritis of the distal radio-ulnar joint J Hand Surg 1991 16B 450 453 10.1016/0266-7681(91)90026-k 1779166

[B5] Tada H Hirayama T Takemitsu Y Extensor tendon rupture after osteoarthrosis of the wrist associated with nonrheumatoid positive ulnar variance Clin Orthop Relat Res 1991 262 141 147 1984911

[B6] Tanaka T Kamada H Ochiai N Extensor tendon rupture in ring and little fingers with DRUJ osteoarthritis without perforating the DRUJ capsule J Orthop Sci 2006 11 221 223 10.1007/s00776-005-0982-9 16568398

[B7] Vaughan-Jackson OJ Rupture of extensor tendons by attrition at the inferior radio-ulnar joint; report of two cases J Bone Joint Surg 1948 30B 528 530 18877990

[B8] Yamazaki H Uchiyama S Hata Y Murakami N Kato H Extensor tendon rupture associated with osteoarthritis of the distal radioulnar joint J Hand Surg 2008 33E 469 474 10.1177/1753193408090098 18687835

